# Current status and needs in the primary healthcare system in Yangon, Myanmar: a mixed-method evaluation

**DOI:** 10.1017/S1463423623000178

**Published:** 2023-05-18

**Authors:** Jihyun Moon, Su Jin Kang, Young Dae Kwon, Eun-mi Song, Jin-Won Noh

**Affiliations:** 1 Graduate School of Medical Sciences, University of Groningen, Groningen, Netherlands; 2 Common Good Partners, Uijeongbu-si, Korea; 3 Institute of Health and Environment, Seoul National University, Seoul, Korea; 4 Department of Humanities and Social Medicine, College of Medicine and Catholic Institute for Healthcare Management, The Catholic University of Korea, Seoul, Korea; 5 Division of Health Administration, Yonsei University Graduate School, Wonju, Korea; 6 Division of Health Administration, College of Software and Digital Healthcare Convergence, Yonsei University, Wonju, Korea

**Keywords:** low- and middle-income countries, mixed methods, Myanmar, primary health care

## Abstract

**Background:**

Many low- and middle-income countries and international organisations have invested resources to strengthen primary health care (PHC). This study aimed to identify the challenges and unmet needs in the current PHC by assessing the experiences and perceptions of healthcare workers in three townships (Htan Ta Pin, Hmawbi, and Taikkyi) in Yangon, Myanmar.

**Methods:**

The study was conducted among healthcare professionals and community leaders in three townships. Adopting a mixed-method approach, a cross-sectional health needs assessment survey was conducted for quantitative data (*n* = 66), and focus group discussions (FGDs) were conducted online for qualitative data.

**Findings:**

Enhancing the management and leadership capacity had the lowest average score on the current achievement (2.81 out of 5 ratings) while strengthening infectious disease control service and accessibility was perceived as the highest mean on the priority of intervention (4.28) and the impact of the intervention (4.7). The FGDs revealed that while specific infrastructures and equipment were reported insufficient and necessary, the need for financial support has been the recurrent theme throughout the discussions.

**Interpretation:**

Utilising the World Health Organisation’s six building block frameworks, our findings suggest that a long-term targeted financial investment in the PHC system is critical in Myanmar through increasing healthcare expenditure per capita.

## Introduction

The Alma-Ata declaration in 1978 stated that primary health care (PHC) is an essential health care being widely accessible to everyone established on socially acceptable, practical, and scientific evidence-based methods and technologies. PHC is at the frontline of the national healthcare system, bridging individuals and communities in continuing healthcare processes with affordable cost with self-reliance and determination (WHO, 1978). This comprehensive and integrated system covers a wide range of resources and areas related to prevention and management (e.g., nutrition, safe water, personal hygiene, maternal and child health, vaccination, quality services, etc.) (Bryant and Richmond, 2017).

The primary healthcare system has been considered a priority of development in low- and middle-income countries (LMICs), reducing inequalities in healthcare delivery by targeting the population in disadvantaged communities. PHC programmes have been widely implemented and adapted to local conditions in LMICs. While PHC has improved the overall population health in those countries, gaps remain in disadvantaged communities (Kruk *et al.*, 2016). The achievement of a comprehensive PHC in LMICs requires country- and community-specific strategies.

The Republic of the Union of Myanmar is a LMIC with a gross national income per capita of US$ 1260 in 2020 (The World Bank, 2021). Myanmar is one of the countries with a critical shortage of healthcare workforce. The basic healthcare staff team of rural health centre located in large villages is responsible for maternal and child health, school health, nutritional promotion, immunisation, community health education, environmental sanitation, disease surveillance and control, treatment of common illnesses, referral services, and training of volunteer health workers. Although the Ministry of Health mobilises healthcare volunteers, including traditional birth attendants, auxiliary midwives, and community health workers at primary and secondary levels in the health system in order to implement PHC, barriers to accessing healthcare services still exist (MIMU, 2017).

Health inequality in Myanmar has worsened across geographic and socioeconomic factors, specifically for those who are in impoverished rural regions. Specifically, the persistent health disparity in household income remains high in the utilisation of maternal healthcare services (Thida *et al.,* 2019). The national data between 2015 and 2016 showed that more than fourfold of pregnant women from urban areas received adequate antenatal care than women from rural areas (Mugo *et al.,* 2020). Despite numerous previous studies assessing the national and community healthcare system in Myanmar, the topics were limited to specific categories in health care (e.g., mental health, oral health, healthcare workforce, etc.), and studies covering a wide range of PHC are also rare (Nguyen *et al.*, 2018; Aung *et al.*, 2019; May *et al.*, 2021). Previous studies on health care in Myanmar used either qualitative or quantitative methods. Since employing a mixed-method approach provides extensive scientific and contextual knowledge (Wisdom *et al*., 2012), this study attempted to provide a comprehensive depiction of the PHC system in the Yangon region by employing both quantitative and qualitative methods.

Thus, this study aimed to identify needs and gaps in the current PHC system in Myanmar by utilising a mixed-method approach of collecting a needs assessment survey and focus group discussions (FGDs) from healthcare workers in three townships in Yangon. In addition, based on the results, this study sought to offer evidence-based structured recommendations by employing the World Health Organisation (WHO) health system building block framework for improving and strengthening the PHC system in resource-limited areas.

## Methods

### Study setting and facilities

**Figure 1.**
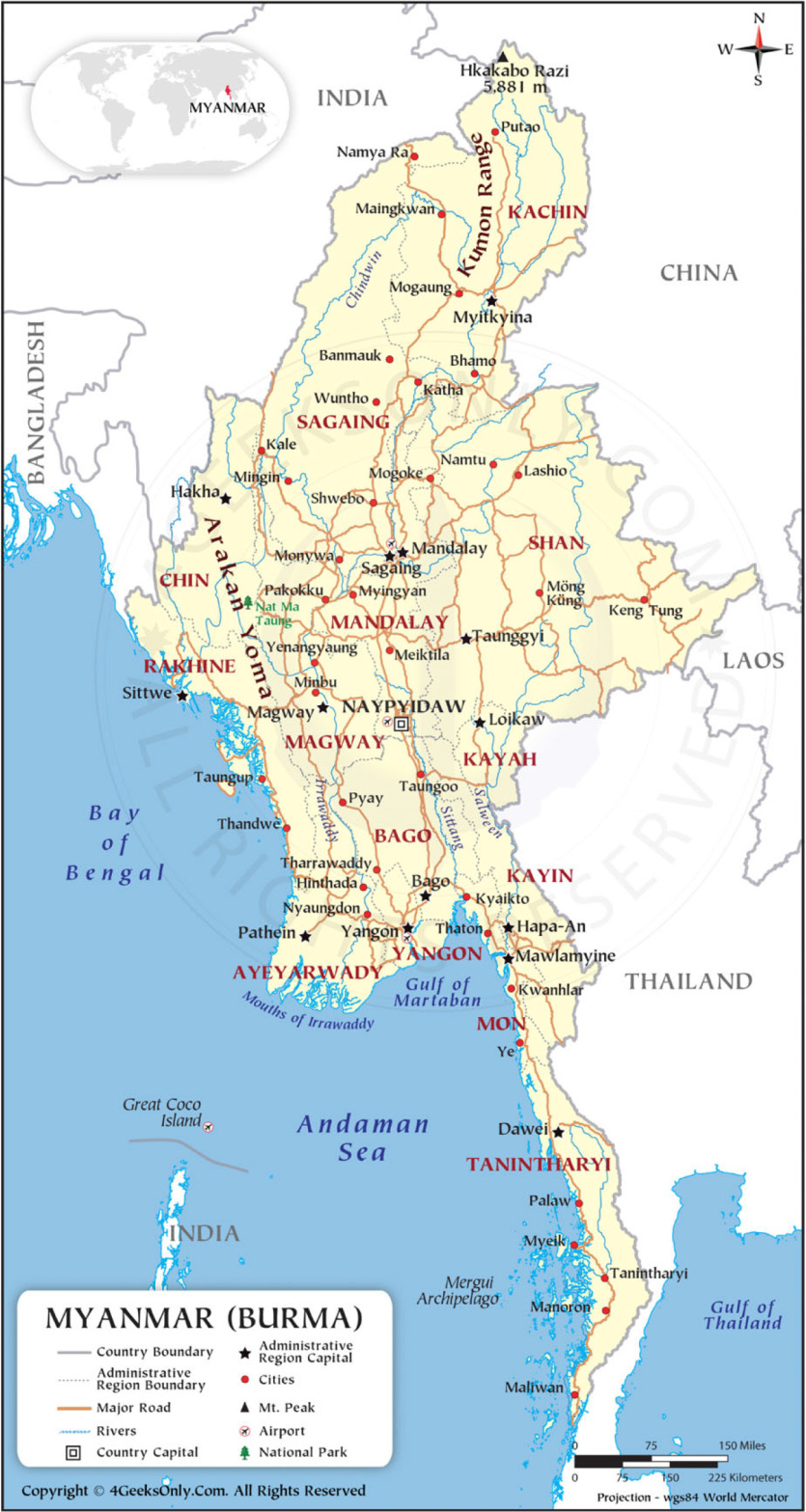
Republic of the Union of Myanmar states and regions with capital cities, 2012^1^.

**Figure 2.**
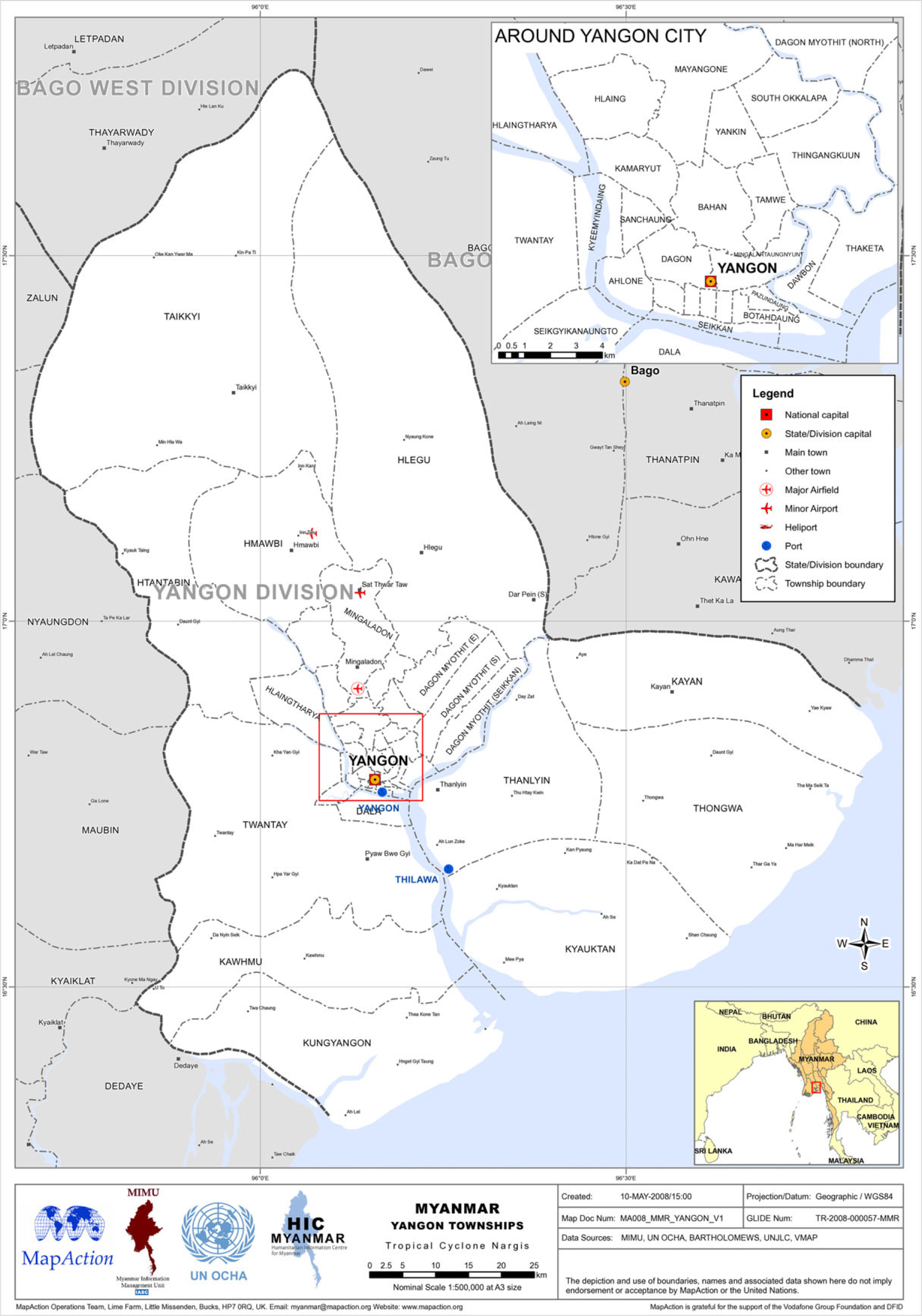
Map of the Yangon region^2^.

Yangon is one of the seven major administrative regions, located in the southern area consisting of 4 districts and 45 townships (Figures 1 and 2). Korea Foundation for International Healthcare (KOFIH) carried out ‘the primary health care system strengthening project (2014–2020)’ focusing on the township of Hlegu, Yangon, Myanmar. KOFIH decided to expand the project to the nearby townships of Hmawbi, Taikkyi, and Htan Ta Pin, along with the successful project results. According to the Township Health Profile (2020), the township of Taikkyi is comparatively larger than the other two townships in terms of the number of facilities. Each township in the area is paired with one township hospital and a few station hospitals. However, the healthcare professionals in those facilities are insufficiently low (Tables 1–3).

**Table 1.** Current status of administrative districts and population by township (2020)^3^

Township	Wards	Village tracts	Number of community leaders	Number of villages	Population	Female population	Male population	Number of women aged 15–49 years
Hmawbi	4	39	39	206	233 920	120 161	113 759	65 353
Taikkyi	20	75	75	462	287 459	140 227	147 232	81 294
Htan Ta Pin	5	55	55	243	175 354	90 154	85 200	48 367
Total	29	169	169	911	696 733	350 542	346 191	195 029

**Table 2.** Status of health and medical personnel by township (2020)^3^

Township	Healthcare service providers (including administrative personnel)							Total
	Doctors	Nurses	Midwives	HA	LHV	PHS1	PHS2	
Hmawbi	4	27	42	7	8	3	39	130
Taikkyi	7	43	59	7	9	3	52	180
Htan Ta Pin	6	26	49	9	5	3	38	136
Total	17	96	150	23	22	9	129	446

HA = health assistant; LHV = lay health visitor; PHS = public health supervisor.

**Table 3.** Number of healthcare institutions by township (2020)^3^

Township	Township Hospitals	Station hospitals	MCH/UHC	RHCs	Sub-RHC	Total
Hmawbi	1	3	1	5	27	37
Taikkyi	1	4	1	4	37	47
Htan Ta Pin	1	5	1	4	35	46
Total	3	12	3	13	99	130

MCH = maternal and child health; UHC = urban health centre; RHCs = rural health centres; Sub-RHC = sub-rural health centre.

A baseline survey was conducted to establish a detailed project plan by investigating the health needs of three townships in the northern region of Yangon. To identify and analyse the gaps between the expected healthcare system and the current ones, we used a mixed-method design, conducting a needs assessment and focus group interviews in three selected townships (Htan Ta Pin, Hmawbi, and Taikkyi) in the Yangon region.

Participants represented by public healthcare providers and local residents were recruited through a purposive sampling method from the three townships. Study participants included healthcare providers: doctors, nurses, midwives, health assistants, public health supervisors and lay health visitors and township medical officers, station medical officers, and school medical officers. Community leaders were also included as representatives of the public. A total of 66 participants from three townships [Htan Ta Pin (*n* = 19), Hmawbi (*n* = 20), Taikkyi (*n* = 27)] participated in the study.

### Study design

This study was cross-sectional using a mixed-method approach. The results from a quantitative needs assessment were complemented by qualitative data. First, the survey questionnaires were completed by healthcare professionals in three townships. The survey instrument was designed by the research team based on the results of the feasibility study. The respondents were asked to rate the current achievement, the priority of intervention, and the impact of the intervention in the following six domains: water, sanitation, and hygiene (9 questions), service of the primary medical centre (10 questions), maternal and child health (6 questions), infectious disease control (5 questions), non-communicable disease control (8 questions), and management and leadership capacity (8 questions). Participants were asked to report their opinion on a 5-point Likert scale.

Second, to explore the perceived gaps and challenges in the PHC system in each township, FGDs were conducted by the participants who responded to the needs assessment. The guidance of semi-structured open-ended questionnaires was utilised (see Table 1 in online supplemental file). FGDs were performed through an online conference tool from 1st January 2021 to 8th due to the ongoing COVID-19 pandemic, and all participants gave informed consent. Participants were asked about their experiences and opinions about the current status of those healthcare services. Each FGD lasted approximately two hours, video-recorded, transcribed, and translated into English.

### Analysis

For quantitative analysis, data collected through the survey were extracted, cleaned, and descriptively analysed, including means of perceived current status, priority, and impact on public health in excel and STATA 17 (StataCorp LP, College Station, Texas). The qualitative data were transferred into excel spreadsheets and analysed using conventional content analysis in each section. Conventional content analysis is generally used to describe phenomena when applicable theory or research literature is limited (Hsieh and Shannon, 2005). In the current study, this analytic approach was utilised to describe the perceived status of healthcare services in three townships in Yangon. The source data were independently reviewed and coded by two researchers (JH and SJ) and discussed the relationship between extracted codes and the results of quantitative data. To better understand the context, researchers contacted the staff who facilitated the FGDs.

## Results

### Characteristics of the participants

Characteristics of study participants are described in Table 4. A total of 66 participants were selected by the research team collaborating with the township health department. There were more female participants (*n* = 44, 66.67%) in the study, and the average age was 43.98 years, and the mean of working experience was 14.23 years.

**Table 4.** Basic characteristics of study population

Township	Htan Ta Pin (*n* = 19)	Taikkyi (*n* = 27)	Hmawbi (*n* = 20)	Total (*n* = 66)
Occupation/position				
Doctor	4	5	2	11 (17.0%)
Nurse	3	5	3	11 (17.0%)
Lady health visitor	1	1	1	3 (4.5%)
Health assistant	2	3	3	8 (12.1%)
Sister	1	2	2	5 (7.6%)
Midwife	4	5	3	12 (18.2%)
Public health supervisor	2	2	2	6 (9.1%)
Community leader	2	4	4	10 (15.1%)
Sex (female)	14 (73.48%)	18 (66.67%)	12 (60%)	44 (67%)
Age	41.74	44.15	46.05	43.98
Years of work experience	12.63	12.42	18.2	14.23

### Six needs assessment domains

Table 5 shows that the average score of the current achievement of six domains had a lower evaluation result overall (mean 2.8–3.2) compared to the perceived priority of the intervention (mean 3.8–4.3) and impact of the intervention (mean 4.1–4.7). This may indicate that respondents consider these six categories to be important to enhance the PHC system, yet they are currently underachieved. More specifically, enhancing management and leadership capacity had the lowest mean (mean = 2.81) and enhancing the non-communicable disease management system had the highest average (mean = 3.17).

**Table 5.** The result of quantitative analysis

Domain	Current achievement	Priority of intervention	Impact of intervention
To increase hygiene-seeking behaviours and awareness at schools and in communities	2.83	3.83	4.19
To strengthen service delivery of primary medical centre and accessibility of public health care in three townships	2.91	3.93	4.26
To strengthen maternal and child health service delivery and accessibility in three townships	3.07	4.17	4.59
To strengthen infectious diseases control service and accessibility	3.05	4.28	4.70
To enhance non-communicable disease management system	3.17	4.25	4.55
To enhance management and leadership capacity	2.81	4.17	4.55

The domains which the key informants perceived positively regarding the impact of the intervention across six domains were strengthening infectious disease control service and accessibility with the highest average score (mean = 4.70) whereas increasing hygiene-seeking behaviours and awareness at schools and communities had the lowest (mean = 4.19). The highest mean score of perceived priority of intervention was strengthening infectious diseases control service and accessibility (mean = 4.28) and increasing hygiene-seeking behaviour and awareness at schools and communities had the lowest (mean = 3.83). The results showed that the key informants in three townships perceived that specific programmes such as infectious diseases, non-communicable disease, and maternal and child health have both higher priority and impact on the community. The other domains, hygiene, the primary medical centre, and leadership/management, in contrast, had lower achievement, were not considered to be the priority, nor have a higher impact on the community.

The following are the results from the analysis of the merged data from quantitative and qualitative surveys under each domain (for the full result, see Table 2 in the online supplemental file).

### Promoting hygiene at schools and in communities

The average score of current achievement of hygiene intervention (mean = 2.83) was the second lowest among the six domains assessed. Also, the hygiene-related intervention was perceived to be the lowest priority (mean = 3.83) and the impact on PHC (mean = 4.19) compared to other interventions. In particular, evaluation of the current status of water, sanitation, and hygiene facilities in public health centres, which include township hospitals, rural health centre, and sub-rural health centre, had the highest average score (mean = 3.11) while water, sanitation, and hygiene facilities in communities had the lowest average score (mean = 2.58). Improving water, sanitation, and hygiene facilities in communities was considered to be prioritised (mean = 4.23) and perceived to have the largest influential (mean = 4.64) intervention in the hygiene domain (see Table 2 in the appendix).

In FGDs, aligned with the result from the needs assessment, most participants shared needs for the improvement of WASH facilities in the community such as accessibility and quality.

‘It is very hard to get water in some villages’. (Community leader, Htan Ta Pin)

‘Concerning with water, sanitation, and hygiene facility, the quality of the water here is not good’. (Health assistant, Hmawbi)

While the current water, sanitation, and hygiene campaigns in communities received a high score, participants perceived that the community has poor awareness of hygiene.

‘Locals don’t know much about water, sanitation, and hygiene care’. (Nurse, Hmawbi)

### Strengthening the primary medical centre service delivery and accessibility

Strengthening the primary medical centre includes a wide range of general necessities at the centre, such as information technology (IT) equipment, transportation, staff education, and funding for necessary costs. Participants scored low on communication equipment (mean = 3.15) and computers with the internet (mean = 2.60). These were also seen as less important in terms of priority (mean = 3.49 and 3.35) and impact (mean = 3.75 and 3.88).

In FGDs, the key informants hardly mentioned IT equipment but emphasised the need for IT education.

‘Most employees from the department of health don’t understand IT and computer-related subjects or have skills’. (Health care assistant, Taikkyi)

In FGDs, all participants except the community leaders claimed the referral system and transportation need the most improvement. Many of them specifically mentioned financial support for emergency cases and overall management and resources for referral.

‘Sending information, basic treatment for villagers in emergency services should be strengthened by the affiliated institution in the future’. (Midwife, Hmawbi)

‘When transferring to other general hospitals, we depend on volunteer associations from Hmawbi township. But they have no good quality training courses on a referral system’. (Township medical officer, Hmawbi)

The FGDs revealed that most low-income families, including migrants, do not seek medical services mainly either due to their work schedule or the burden of transportation expenses.

‘As you know, we don’t need to pay a lot for medical treatment at the primary medical centre, but many villagers cannot afford transportation charges’. (Community leader, Htan Ta Pin)

‘When we visit the mobile clinic, we often do not see the local patients because many of them went to their work for a living during the daytime’. (Township medical officer, Hmawbi)

‘Migrant workers are unable to see the doctor in the primary medical centre due to financial difficulty’. (Nurse, Taikkyi)

### Strengthening maternal and child health service delivery and accessibility

Strengthening maternal and child health was scored as the second highest in current achievement (mean = 3.07) and would have the largest impact (mean = 4.59) among the assessed domains. Specifically, providing essential healthcare services for mothers (mean = 4.85) and services for children (mean = 4.66) was viewed as having the highest impact, and current services were scored (mean = 3.67) high. Also, while providing kits and allowances (e.g., transportation) for mothers was perceived to have a high impact (mean = 4.74 and 4.78) on PHC, their current status was scored poorly (mean = 2.89 and 2.37).

Likewise, participants in FGDs mentioned a lack of supplies, transportation, and financial support, as well as the need for staff training for maternal and child health.

‘In the labour room, there is no blue photo, no warmer. There is no light in maternity wards’. (Township medical officer, Htan Ta Pin)

‘We need refreshing training courses for antenatal care and training courses for postnatal care for midwives’. (Midwife, Hmawbi)

‘The majority of migrant pregnant women do not take good care of their health. They usually come to the clinic when they are about to give birth because they come to Yangon for a living’. (Township medical officer, Taikkyi)

### Strengthening infectious disease control services and accessibility

Respondents evaluated the current status of infectious disease control as relatively high for medicines (mean = 3.46) and equipment (mean = 3.19) but low for reserve budget (mean = 2.31) and training for the community (mean = 3.13). They perceived that securing a budget for an imminent outbreak and seasonal disease control (mean = 4.34) and training for the locals (mean = 4.45) should be prioritised.

‘Health education is very important and I would like international non-governmental organisations and non-governmental organisations to give us more information on water, sanitation, and hygiene, infectious diseases and other diseases’. (Community leader, Taikkyi)

‘Usually people affected from all of these diseases, including infectious diseases, can be admitted to the hospitals but they are not well-equipped for treatment’. (Community leader, Taikkyi)

‘The best way to prevent infectious and non-infectious diseases is to educate the residents and support them with equipment’. (Nurse, Taikkyi)

The findings of the analysis suggested that staff training and public education for infectious diseases were the predominant needs.

### Enhancing the non-communicable disease management system

The electronic health statistics system, the least developed at the current stage, was scored the second top priority though being perceived not to have a higher impact than other resources such as medical equipment. Also, supporting clinic services and nutrition for the elderly at rural health centre was viewed as having a higher priority (mean = 4.58) while its current status was rated (mean = 3.37) higher than other services.

In the FGDs, participants often addressed the issue of not having enough non-communicable disease drugs to provide for the community members. Non-communicable disease showed challenges in the management of medication, thus expressing the need for developing an electronic registration system.

‘We have difficulty in controlling noncommunicable disease, specifically providing medication. Some drugs are sufficiently supported by the Ministry of Health while for some we have to buy them outside’. (Nurse, Taikkyi)

### Enhancing management and leadership capacity

Improving management and leadership scored high on its priority (mean = 4.17) and impact (mean = 4.55) but the lowest rating in the current status (mean = 2.81). In particular, the current financial support for transportation for travelling and telephone fees received the worst rating (mean = 2.51). While financial support was perceived as having less impact (mean = 4.57) than other items (e.g., study tour, best health worker award), this scored as the second top priority of the intervention (mean = 4.48).

In the FGDs, midwives and nurses shared their working conditions that they often paid for travel expenses on their own and some required leadership training. Most of the issues in FGDs were about expenses which they perceived as management and leadership problems.

‘If possible, we would like to get travel funds when we have to visit clinics to provide care’. (Midwife, Htan Ta Pin)

‘The workload is high and getting transportation is difficult. I have to cover my own expenses’. (Midwife, Taikkyi)

## Discussion

The current study utilised a mixed-method evaluation, conducting a needs assessment survey and FGD with the frontline healthcare workers and community leaders to gain a comprehensive understanding and knowledge of the current condition in PHC in the Yangon Region, Myanmar. Our survey results showed that elements related to the triple burden of diseases: infectious diseases, maternal and child health, and non-communicable diseases (Grundy *et al.*, 2014) overall received higher current achievement scores while hygiene, the primary medical centre, and management received a relatively lower average rating. The FGD revealed significant findings that most of the discussions were centred around infrastructure-related matters in all domains, indicating those domains that received lower scores – hygiene, primary medical centre, and leadership/ management – were evaluated considering infrastructure availability and implementation. Our finding demonstrates that infrastructure in lieu of health services is perceived as the fundamental basis of the primary healthcare system.

While rigorous and robust evaluation and/or structured recommendations are necessary for developing and investing in the national healthcare system, no previous study in Myanmar has provided such guidance. In this regard, we utilised the WHO’s health system building block framework to construct the assessed priority areas, fitting to the current and future challenges and discussing recommendations and future directions for strengthening the PHC system in Myanmar (Table 6).

**Table 6.** The average score of primary healthcare system

Six building blocks	Current achievement	Priority of intervention	Impact of intervention	The degree of necessity
Service delivery (*n* = 19)	3.00	3.99	4.34	0.99
Medication and technology (*n* = 9)	3.24	4.05	4.46	0.81
Information system (*n* = 3)	2.80	4.00	4.23	1.20
Human resource (*n* = 4)	2.86	3.92	4.35	1.06
Financing (*n* = 5)	2.41	4.43	4.74	2.02
Governance and leadership (*n* = 6)	3.00	4.22	4.57	1.22

### WHO’s six building block frameworks

The WHO’s health system building block is a framework for action to address the urgent need for the performance of the health system (WHO, 2010). The core six components of building blocks are a) service delivery, b) human resources, c) medication and technologies, d) health information systems, e) financing, and f) governance and leadership. The current average achievement scores were subtracted from the average priority of intervention scores to indicate the performance gap/degree of necessity. Then, we selected the top four components of six building blocks that have the subtracted performance gap above 1.00: financing, governance and leadership, information system, and human resource. This framework is designed to help in guiding policymakers to identify the urgent needs and strengthen the healthcare system (Shakarishvili *et al.*, 2011) as well as to provide researchers with systems thinking to address broad and complex issues and to action research (Savigny *et al.*, 2009).

### Financing

Financing showed the least achievement but the highest priority and impact of the intervention. Given that financing can restrain the improvement of other building blocks, the government must secure healthcare financing to help people receive necessary health services as well as for healthcare workers to receive proper training and incentives (WHO, 2007).

Despite the increase in the budget allocation for health to 3.40% of total government expenditure in 2014–2015 from the 1.00% in 2010–2011, Myanmar still has the lowest health expenditure in the Asia-Pacific region. The most recent level has decreased, accounting for 4.79% of the total GDP in 2018 (The World Bank, 2021; WHO, 2014). Since the military holds the largest share of the national budget (40.0%), the major contributing factor to the weaker growth, the Myanmar government should shift its focus to redistributing government revenues, increasing revenue mobilisation, specific goods or services, and voluntary sources of revenue (Loyer *et al.*, 2014; Elovainio and Evans, 2017). Improving the efficiencies of resources in health outcomes also requires a political will to tackle existing corrupt practices and inefficacy in medicine procurement and use, and distribution (Elovainio and Evans, 2017).

### Leadership and governance

While leadership and governance are hinged on the context, capacity, information, and the spectrum of actor involvement, the overarching principle can be observed through the experience, including policy guidance, intelligence and oversight, collaboration and coalition building, regulation, system design, and accountability (WHO, 2007; Naher *et al.*, 2020). Our study revealed only the partial aspects of leadership and governance – health programme management and human resources – since the participants were the frontline healthcare workers, holding limited experiences and views about the governance system. Also, the support system and engagement of stakeholders in the local community were perceived as weak in the Yangon region so the development of a well-designed leadership and support programme must be considered.

### Health information system

A well-established health information system requires the utilisation and exchange of reliable and timely health information at different levels of the PHC system (WHO, 2007). Also, population and facility-based data with the capacity to detect, investigate, communicate, and contain events that threaten public health security, synthesise the collected information, and promote the availability of the knowledge are necessary components of a well-functioning information system (WHO, 2007). In the Yangon region, however, public health facilities lack data or information on certain groups, such as migrant workers, resulting in a widening health equity gap. Also, our findings indicate that public health facilities have far inadequate information systems on medical products including essential medicine. Several study participants complained about the mismatches between the supply and demand of non-communicable disease medicine, implying a poor information system for medication management. In this regard, the Ministry of Health and Sports in Myanmar is currently developing an electronic information system and putting the effort into the overall readiness for this technology (Oo *et al.,* 2021).

One of the core focuses of the health information system building block is technology-related education and knowledge that comprehensive on-the-job training and contextualising and localising the curriculum in training programmes need to be developed and regularly updated (Oo *et al.,* 2021).

### Human resources

Most healthcare workers in the Yangon region are either unpaid lay volunteers or paid professionals. Thus, managing labour markets and improving the distribution and operating system are critical to achieving a well-performing healthcare workforce (WHO, 2007). The PHC workers in this region, however, faced the problems of the healthcare workforce shortage and unbalanced workforce distribution over the areas and health programmes. The report of 2016 showed that the number of medical doctors in 13 states (out of 15) is below the WHO’s recommended minimum number (1 per 1000 population) (Saw *et al.*, 2019). Particularly, the shortage of human resources for public health was described due to lower remuneration, unstable policy, and uneven workload (Saw *et al.*, 2019). Limited skill-based training affected the performance of health providers as well as had an impact on recruiting and maintaining the workforce. One study found a strong need for specific training regarding maternal and neonatal health, communication between professionals and lay volunteers, and health education for the health professionals (Mosca *et al.,* 2020). The human resources building block strongly suggest collaborating with other government sectors and ministries such as educational, economic, and rural developmental sectors.

Our findings through the building block approach correspond to other studies of Myanmar (Sommanustweechai *et al.*, 2016; Ei *et al.*, 2019; Oo *et al.,* 2021). One study reviewed the oral healthcare system in Myanmar using six building blocks and found low utilisation of oral healthcare services, lack of workforce, inadequate budget with non-transparent expenditure, and insufficient supply (Saw *et al.*, 2019). Our evidence indicates that the fundamental issues in the healthcare system crosscut a wide range of health care. Despite the financial issue appearing to be an overarching problem in this study, the scheme of increasing national funding alone cannot address challenges, especially at the township level due to varied context conditions (Htun *et al.,* 2021). Hence, the implementation of targeted intervention should be proceeded with identifying context-specific barriers and facilitators to optimise the effectiveness. Likewise, numerous studies on other LMICs have encountered similar issues in their PHC system: a lack of resources, accessible services and workforce, insufficient funding, and the disparity between urban and rural areas in resource-restricted settings (Makwero, 2018; Nang *et al.*, 2019; Tang and Zhao, 2019; Moalong *et al.*, 2021). Poor coordination, inequity in deployment policies, and ethnic conflicts were the contributing factors worsening these issues (Makwero, 2018; Tang and Zhao, 2019).

While findings provide limited details in the process of occurring challenges, the current study contributed to the understanding of the healthcare system in the Yangon region in Myanmar through mixed methods with empirical data. The practical implication of this study contributes knowledge to guide and develop proper and sustainable interventions, reflecting the needs and interests of the community provided by the front-level stakeholders. It is to note that the survey in this study was conducted by an INGO that recruiting sufficient local community representatives was limited, which might have caused potential information bias in the findings. Also, due to internal conflict and the COVID-19 pandemic, the current study was conducted with limited resources and time constraints, unable to reach out to local staff for further questions. Yet, this study finds sufficiently similar needs and challenges in three townships. Diverse voices from the community and partners such as volunteers ought to be considered in the improvement of the PHC system in Myanmar. Lastly, programmes in each domain need to be routinely evaluated to account the changing circumstances and needs of the community, and the political dynamics and challenges of the country.

## Conclusion

This study highlighted multiple features of the PHC environment in the Yangon regions in Myanmar. The insights gained from healthcare workers at the local site would provide invaluable context and guidance in building and strengthening the PHC system as well as in providing a foundation for policy and practice improvements across the regions in Myanmar. The PHC system is fundamental for fulfilling health-related Sustainable Development Goals. While much advancement and improvement have been made in the PHC system and population health in Myanmar, the current study demonstrated insufficient financial support and management in infrastructure, equipment, medication, healthcare personnel, transportation, and the individuals in poverty. The results also indicate that these considerable impediments to getting necessary health services disproportionately impact those living in geographically remote areas, exacerbating health disparities. The implication of this study thus is investing in the PHC system by increasing health expenditure to improve the health outcomes of people in Myanmar. The government should increase fund allocation to PHC, specifically expanding services and improving the service quality through investing to train skilled healthcare staff. Also, prioritising community demand and need as well as integrating perspectives from the community members is critical to empowering the individuals and communities. Lastly, the ongoing research should continue to identify and address barriers and insufficiencies in the PHC system in Myanmar to make evidence- and context-based decisions and design a sustainably effective PHC system at all levels.

## Footnotes

1 BurningCompass.Com, 2023. Myanmar Map. [Online]. Available from: https://www.burningcompass.com/countries/myanmar/myanmar-map.html

2 MapAction, MIMU, and OCHA, 2008. Reference map of Yangon Townships, Myanmar. [Online]. Available from: https://reliefweb.int/map/myanmar/reference-map-yangon-townships-myanmar

3 Township Health Profile, Hmawbi Township, Taikkyi Township, Htantabin Township, Yangon Region 2020.

## Supplementary material

For supplementary material accompanying this paper visit http://doi.org/10.1017/S1463423623000178. click here to view supplementary material
