# Supplementary material for: Current status and needs in the primary healthcare system in Yangon, Myanmar: a mixed-method evaluation
Source: Prim Health Care Res Dev. 2023 May 18;24:e37. doi: 10.1017/S1463423623000178 (PMC10227472; doi:10.1017/S1463423623000178)
Supplement: Supplementary file 1 [file S1463423623000178sup001.docx]

**Table 1. Questionnaire for focus group discussion**

| **Question type** | **Questions** |
| --- | --- |
| Introduction | Please tell us about the characteristics of the region.  Overall, how would you rate the health status of your community, excellent, very good, good, fair or poor? Why do you say that? |
| Transition | Please think of the current primary health care system in your community. |
| Key | How many residents does the institution provide health care services to?  On average, how many residents visit each day?  What services do you provide to residents?  Please tell us if residents have any difficulties using ______________ |
|  | The situation of village health committees  The structure, management, service delivery, skills and performance of health staff  The situation of community’s maternal and child health care services  The situation of community’s water, sanitation, and hygiene care services  The situation of community’s non-communicable diseases  The situation of community’s infectious diseases |
|  | Please tell us if you have any support necessary to provide better service to residents  What types of services should be strengthened by the affiliated institution in the future?  What do you think would improve the primary health care system of the community? |
| Ending | Do you have any suggestions or feedback you would like to share? |

**Table 2. The average score of primary health care system in three townships**

| **Six Building Blocks** | **Categories** | **Current achievement** | | | **Priority of intervention** | | | | **Impact of intervention** | | | | |
| --- | --- | --- | --- | --- | --- | --- | --- | --- | --- | --- | --- | --- | --- |
|  |  | Htan Tan Pin (n=19) | Taikkyi (n=26) | Hmawbi (n=20) | | Htan Tan Pin (n=19) | Taikkyi (n=26) | Hmawbi (n=20) | | Htan Tan Pin (n=19) | Taikkyi (n=26) | Hmawbi (n=20) | |
| Service delivery | [WASH] Improve WASH facilities of communities | 2.58 | 2.15 | 3.00 | | 4.00 | 4.53 | 4.15 | | 4.89 | 4.69 | 4.35 |  |
| Financing | [PMC] Support patient referral by providing (transportation) funds for emergent cases | 3.05 | 2.23 | 2.60 | | 4.84 | 4.77 | 4.65 | | 4.58 | 4.50 | 5.00 |  |
| Financing | [PMC] Support low-income families to access by providing health equity fund | 2.79 | 2.35 | 1.90 | | 4.26 | 4.61 | 4.25 | | 5.00 | 4.54 | 4.55 |  |
| Service delivery | [MCH] Provide mother kits for mothers who give a birth in facilities | 3.37 | 2.96 | 2.35 | | 4.00 | 4.19 | 5.00 | | 4.94 | 4.27 | 5.00 |  |
| Financing | [MCH] Provide allowances (including transportation) for mothers who give a birth in facilities | 3.37 | 1.85 | 1.90 | | 4.00 | 4.65 | 4.45 | | 4.79 | 4.54 | 5.00 |  |
| Human resources | [Infectious disease] Provide infectious diseases training for community (local residents) | 4.00 | 2.85 | 2.55 | | 4.95 | 4.50 | 3.90 | | 5.00 | 4.46 | 4.44 |  |
| Financing | [Infectious disease] Reserve budget for emergent outbreak & seasonal disease control | 3.21 | 1.96 | 1.75 | | 4.26 | 4.11 | 4.65 | | 5.00 | 4.65 | 5.00 |  |
| Information systems | [NCD] Build health statistic system through electronic-based patient registration | 3.74 | 1.65 | 2.15 | | 4.74 | 4.65 | 4.05 | | 4.95 | 4.61 | 4.15 |  |
| Human resources | [Leadership] Support best health workers selection and study tour | 3.53 | 2.73 | 3.65 | | 4.37 | 4.31 | 4.40 | | 4.95 | 4.92 | 4.50 |  |
| Financing | [Leadership] Provide incentives for staff who gives service for remoted local clients | 3.74 | 1.65 | 2.15 | | 4.74 | 4.65 | 4.05 | | 4.94 | 4.61 | 4.15 |  |
| WASH: water, sanitation, and hygiene, PMC: primary medical centre, MCH: maternal and child health, NCD: non-communicable diseases | | | | | | | | | | | | |  |
